# Design of Superlubricity System Using Si_3_N_4_/Polyimide as the Friction Pair and Nematic Liquid Crystals as the Lubricant

**DOI:** 10.3390/polym15183693

**Published:** 2023-09-07

**Authors:** Xinlei Gao, Yuwei Cheng, Miaomiao Shi, Hao Chen, Li Wu, Tingting Wang

**Affiliations:** 1 School of Materials Science and Engineering, Hubei University, Wuhan 430062, China; 2 School of Chemical and Environmental Engineering, Wuhan Polytechnic University, Wuhan 430023, China; yw_123cheng@163.com (Y.C.); m17862020903_1@163.com (M.S.); tt_wang88@163.com (T.W.); 3 School of Chemistry and Environmental Engineering, Wuhan Institute of Technology, Wuhan 430074, China; wu88888li@163.com

**Keywords:** superlubricity, polyimide, Si_3_N_4_, liquid crystal, alignment

## Abstract

Polyimide (PI) is a high-performance engineering plastic used as a bearing material. A superlubricity system using Si_3_N_4_/PI as the friction pair and nematic liquid crystals (LCs) as the lubricant was designed. The superlubricity performance was studied by simulating the start-stop condition of the machine, and it was found that the superlubricity system had good reproducibility and stability. In the superlubricity system, friction aligned with the PI molecules, and this alignment was less relevant compared to which substance was rubbing on the PI. Oriented PI molecules induced LC molecule alignment when the pretilt angle was very small, and the LC molecules were almost parallel to the PI molecules due to the one-dimensional ordered arrangement of LC molecules and low viscosity, which is conducive to the occurrence of the superlubricity phenomenon.

## 1. Introduction

Polyimide (PI) is a class of heat-resistant organic polymers developed in the 1950s. PI can be used for a long time in a temperature range from −200 to 300 °C, with a thermal decomposition temperature of 600 °C, and it is one of the most thermally stable organic polymers [1]. As a high-performance engineering plastic, PI has excellent mechanical properties, radiation resistance, good tribological properties and processability and is commonly used as bearing materials, such as high-speed rolling bearing cages or gyro motor bearings with oil cages. Therefore, the study of friction properties in PI has been a hot issue in tribology. One useful way to solve friction problems is to provide an effective lubrication system. With the rapid growth of the global industry, one of the most effective ways to increase productivity and reduce energy consumption is to use high-performance lubrication systems. Among these, the design and development of new lubrication systems have been a hot issue in academia and the industry. As a new field of tribology, the design and construction of superlubricity systems have attracted particular attention.

Superlubricity refers to the relative sliding between two surfaces in contact with each other with a friction coefficient on the order of 0.001 or less and is usually categorized into two types: solid superlubricity and liquid superlubricity. In terms of solid superlubricity, Zheng’s team conducted a series of superlubricity experiments using the “self-retraction” phenomenon of graphite and realized the macro- and microscopic stabilization of superlubricity [2,3,4] with the friction coefficient reduced to less than 0.0001 and the load-carrying capacity reaching more than 2 G Pa [5]. Zhang’s team systematically carried out engineering-oriented solid superlubricity research and made an outlook on this future direction [6]. Liquid superlubricity is subject to far fewer environmental constraints than solid superlubricity. Researchers have found that many different kinds of liquids, such as pure water [7,8,9,10,11,12,13], acidic aqueous solutions [14,15,16,17], ionic liquids [18,19,20,21], oil-based systems [22,23,24,25,26,27,28,29], liquid polymers [30,31], as well as liquid lubricants with some nanomaterials (graphene, etc.) [32,33] can exhibit superlubricity properties at the macroscopic scale under specific conditions. Moreover, much progress has been made in the study of liquid superlubricity mechanisms. It is widely recognized that the key to achieving liquid superlubricity is to ensure that the liquid molecules are stabilized on the surface of the friction pair under external pressure while providing very low shear strength [34]. Through the initial exploration of liquid superlubricity systems, our team also discovered a superlubricity system using nematic liquid crystals (LCs) as the lubricant and GCr15 steel with PI as the friction pair [35,36].

Liquid crystal is an intermediate phase between a solid and a liquid, and at a certain temperature range, it presents a special material state different from a solid, liquid, or gas. It is characterized by both the molecular arrangement and anisotropy of crystals and the fluidity of liquid; therefore, the LC phase is also known as the mesocrystalline phase. Nematic LCs are thermotropic LCs; they are one-dimensionally ordered phases with the long axes of molecules basically aligned parallel in one direction. Nematic LCs are mostly rod-like structures, which are easy to orient and have been studied more in the field of lubrication. However, some typical thermotropic LCs have a phase transition temperature range (from the melting point to the clearing point) of only a few tens of degrees, which limits their applications. Therefore, in practice, mixtures of low co-melting point broad-temperature nematic LCs consisting of multiple LCs are usually used. Interestingly, PI is one of the most commonly used materials for LC alignment. In LC displays, PI, as the most industrially used LC-aligned material, is made into a thin film layer (known as the orientation layer), which is in direct contact with LCs and serves to induce the LC molecules to arrange on the surface of the orientation layer in a certain direction and angle [37]. A PI film is formed between the substrate housing the LCs, and the PI controls the pretilt angle of the LCs so that the LCs are oriented in a certain direction. In industrial production, the rubbing alignment is a relatively simple and stable method of controlling the orientation of LCs with PI. Scratches are left on the PI film by rubbing to form grooves, which creates a certain anchoring force between the LCs and the PI; then, the LCs are aligned along the grooves and oriented toward the rubbing direction in their natural state. A fixed pretilt angle can be formed between the LCs and the PI. The structural characteristics of PI and LCs affect the pretilt angle.

In a previous study, we tested a system with a PI film and GCr15 steel as the friction pair and nematic LCs as the lubricant and found that friction led to the alignment of PI, and aligned PI induced the orientation of LCs in the grooves of PI wear scars, resulting in the stable superlubricity property of the system [35,36]. For this type of superlubricity system, the alignment of PI and LCs is very important. GCr15 steel might play a role in providing friction behavior, and we could try to use other friction parts instead of GCr15 steel for similar tests. In this paper, silicon nitride (Si_3_N_4_) was chosen to replace GCr15 steel as a friction part in this study. Si_3_N_4_ has many advantages, such as low density, high hardness, high-temperature resistance, corrosion resistance, electrical insulation, non-conductivity, high compressive strength, and good self-lubricating properties.

Silicon nitride is also an important bearing material. As we all know, bearings are important core basic parts in contemporary mechanical equipment, and the performance of bearings directly determines the operational reliability of various types of equipment [38]. The main function of bearings is to support mechanical rotating bodies, reduce the friction coefficient during their movement and ensure their rotational accuracy. For bearings, good lubrication is the key to reducing friction and wear between the friction parts and improving the operation’s accuracy. In fact, friction control in the bearing systems is very important. Once the friction becomes controllable, or even superlubricity occurs, it is better able to guarantee the high precision, sensitivity and long life of the mechanical operation. Si_3_N_4_ as a bearing material is characterized by low-density, high-limiting speed, low-friction torque, good running accuracy, long service life, excellent self-lubrication, and good anti-wear and corrosion resistance. In particular, due to the insulating properties of Si_3_N_4_, it can play a role in reducing current corrosion in various generator bearings, which can be used as bearings for new energy vehicles, wind power generation equipment, medical diagnostic equipment with magnetism, and semiconductor equipment. Si_3_N_4_ bearings are one of the hottest and best-performing high-end ceramic bearings available.

Although the unique material properties of LCs, PI and Si_3_N_4_ have attracted attention in the field of tribology and have been researched and applied accordingly, there is no systematic research on the friction system with nematic LCs as a lubricant and PI and Si_3_N_4_ as the friction pair. Therefore, the aim of this work is to investigate the friction behavior of the system with nematic LCs, PI and Si_3_N_4_, to provide fundamental tribological data for the design and development of bearings using PI, and to further complement the research of superlubricity behavior via PI-induced alignment.

## 2. Materials and Methods

Friction tests were carried out using a UMT-3 Microtribometer (CETR, CA, USA). During the test, the room temperature was 25 °C. The test ball (4.76 mm in diameter, including GCr15 steel, Si_3_N_4_, or silicon carbide (SiC)) was used as a static specimen, and the PMDA (pyromellitic dianhydride)-ODA (4,4′-oxybisbenzenamine) PI film was affixed to a stationary disk as a disc specimen of rotational motion, rotating clockwise at the corresponding speed, with an annular rubbing path and a radius of 8.5 mm. This load was applied vertically through the centerline of the ball specimen, and the test was conducted in either a point-plane or plane-plane contact mode. While applying the corresponding load, 0.1–0.2 mL of LCs was added as a lubricant between the test ball and the PMDA-ODA PI film. During the test, the computer automatically recorded the friction coefficient.

**Figure 1:**
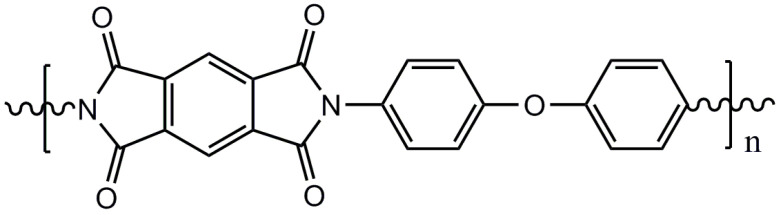
Molecular structure formula of PI.

A total of seven kinds of LCs were involved in the test, and their molecular structures are shown in Table 1. GCr15 steel balls were selected from NSK’s 51103 bearing steel balls (NSK Ltd., Tokyo, Japan). Si_3_N_4_ balls were produced by Wuhan Yuanda Bearing Co. (Wuhan, China). SiC balls were supplied by Shanghai Fanlian Technology Co. (Shanghai, China). The PI film was a commercial PMDA-ODA PI film (Kapton) produced by DuPont (Wilmington, DE, USA) with a thickness of 0.127 mm, whose molecular structure formula is shown in Figure 1.

The above seven single-molecule LCs were mixed to form nine mixed LCs (at a room temperature liquid crystalline state) and the mixed LCs were all compounded with 5CB as the main component, and added with different mass fractions of 3UTPP2, 3UTPP4, 4UTPP3, 5CEPO2, 2CEPPN, or 3CEPC3. In order to distinguish the mixed LCs, mixed LCs are named uniformly in this paper. The letters ABC are used to name the mixed LCs with different concentrations, where A stands for high and low concentrations of mixed LCs with mass fractions of 80% and 20%, respectively; B stands for high and low concentrations of mixed LCs with mass fractions of 85% and 15%, respectively; and C stands for high and low concentrations mixed with LCs and mass fractions of 90% and 10%, respectively. The Arabic numerals 1–6 following these letters represent the subcomponents 3UTPP2, 3UTPP4, 4UTPP3, 5CEPO2, 3CEPC3, and 2CEPPN of the mixed LCs, respectively. The specific composition and formulation of the mixed LCs are shown in Table 2.

### 2.1. Friction Test

#### 2.1.1. Optimal Lubricant Screening

Tribological tests were carried out with 5CB or the nine mixed LCs as lubricants, with Si_3_N_4_/PI (PMDA-ODA) as the friction pair, at a load of 5 N and a rotation speed of 200 rpm (177.92 mm/s) for a period of 3600 s for each circle of tests, with point–plane contact. The mixed LCs with the best friction-reducing performance were screened as the lubricant in subsequent studies.

#### 2.1.2. Friction Tests of the Optimal Lubricant

The lubricant used in this test was the lubricant selected in Section 2.1.1.

##### Point-Plane Contact

(a) Different loads

The test was conducted at 200 rpm (177.92 mm/s) for 3600 s per circle, with an initial load of 5 N, followed by an increase of 10 N per circle until the PI film ruptured and the test was stopped.

(b) Different rotation speeds

The test load was 5 N, and the test time was 3600 s per circle, with an initial speed of 50 rpm (44.48 mm/s), which then increased by 50 rpm per circle up to 400 rpm (355.84 mm/s).

(c) Long-time test

The test load was 5 N, the speed was 200 rpm (177.92 mm/s), and the duration of each circle of tests was 3600 s to simulate the starting and stopping of the machine. During each circle of testing, the machine was stopped and then reloaded for 10 consecutive circles.

##### Plane–Plane Contact

Since the contact mode of the plain bearing is face contact, in order to simulate the practical application, we polished the flat surface on the Si_3_N_4_ ball and conducted friction tests between the flat surface of the Si_3_N_4_ ball and the PI film in the face contact mode. Si_3_N_4_ ball plane grinding was performed using a microtribometer (UMT-3). A Si_3_N_4_ ball (4.76 mm in diameter) was used as a static specimen (upper specimen). Under the no-lubrication condition, 180-mesh sandpaper was selected as the friction part, and this sandpaper was attached to the surface of a smooth metal disk, which was rotated at a speed of 60 rpm, with an annular friction path radius of 8.5 mm and a load of 10 N, with point–plane contact for 8 min. The Si_3_N_4_ ball was then rotated 90° clockwise, and the operating conditions were kept constant for 8 min. Then, the Si_3_N_4_ ball was rotated 180° counterclockwise and replaced with 2000-mesh sandpaper, and the other operating conditions remained unchanged for 5 min. Finally, the Si_3_N_4_ ball was rotated 90° counterclockwise, and the other conditions were kept constant for 5 min to obtain a flat surface on the Si_3_N_4_ ball.

The obtained Si_3_N_4_ plane was placed in plane–plane contact with the PI film at a test load of 5 N and a rotation speed of 200 rpm; the duration of each circle of tests was 3600 s, which still stimulated the starting and stopping of the machine, and this machine would be stopped and reloaded for each circle, which ran for 8 consecutive cycles.

### 2.2. Variation in Friction Part

In order to further investigate the correlation between the superlubricity phenomenon and the material of friction pair in this friction system, we replaced the Si_3_N_4_ ball with a GCr15 steel ball or SiC ball. The flat surfaces of the GCr15 steel ball and the SiC ball were prepared separately, and similar operations were carried out, as described in Section 2.1.2 for plane-plane contact (the grinding conditions were not identical due to the different hardness of the materials). Plane–plane friction tests were conducted using GCr15/PI or SiC/PI as the friction pair and the optimal LCs as the lubricant.

#### 2.2.1. Grinding of GCr15 Steel Plane or SiC Plane

##### GCr15 Steel Plane

A GCr15 steel ball (4.76 mm in diameter) was used as the static specimen (upper specimen). In the absence of lubrication, 180-mesh sandpaper was first selected as the mating pair to be attached to the smooth surface of the metal disc, and the attached sandpaper was rotated at a speed of 60 rpm, with the radius of the circular friction path at 8.5 mm, and a load of 5 N. The run was carried out for 5 min with point–plane contact. The GCr15 steel ball was then rotated 90° clockwise and ran for 5 min under the same test conditions. The GCr15 steel ball was again rotated 90° counterclockwise to return to the initial position, 1000-mesh sandpaper was replaced, other conditions remained unchanged, and the machine ran for 3 min. Finally, the GCr15 steel ball was rotated 90° clockwise with 2000-mesh sandpaper; the other test conditions were kept constant while the machine ran for 5 min to obtain a plane on the GCr15 steel ball.

##### SiC Plane

The SiC plane was obtained in a similar way to the Si_3_N_4_ plane and GCr15 steel plane, using a SiC ball (4.76 mm in diameter) as the static specimen (upper specimen). In the absence of lubrication, 180-mesh sandpaper was selected and attached to the smooth surface of the metal disc, which rotated at a speed of 60 rpm, with a friction radius of 8.5 mm and a load of 20 N. The point–plane contact operated for 5 min. The SiC ball was then rotated 90° clockwise and ran for 5 min under the same conditions. The SiC ball was again rotated 90° counterclockwise to return to its initial position, 1000-mesh sandpaper was replaced, and the other conditions remained unchanged for 3 min. Finally, the SiC ball was rotated 90° clockwise with 2000-mesh sandpaper, and the other conditions were maintained for 3 min to obtain a plane on the SiC ball.

#### 2.2.2. Plane-Plane Contact Friction Test of GCr15/PI or SiC/PI System

The obtained GCr15 steel plane or SiC plane, respectively, was contacted with the PI film plane and subjected to friction tests under the same test conditions as Section 2.1.2 for plane-plane contact.

### 2.3. Surface Analysis of PI

#### 2.3.1. Surface Topography Analysis

The surfaces of the rubbing PI in Section 2.1.2 for plane-plane contact and Section 2.2.2 were analyzed by a white-light interferometer (AE-100M).

#### 2.3.2. Surface-Enhanced Raman Scattering Spectra Analysis

Silver mirror tests [36] were performed on the original PI film and the PI films subjected to the plane–plane contact tests described in Section 2.1.2 for plane-plane contact and Section 2.2.2, respectively. Surface-enhanced Raman scattering (SERS) spectroscopic studies were carried out on silver-coated PI film samples using a laser confocal micro-Raman spectrometer (DXR).

## 3. Results and Discussion

### 3.1. Friction Test

#### 3.1.1. Optimal Lubricant Screening

**Figure 2:**
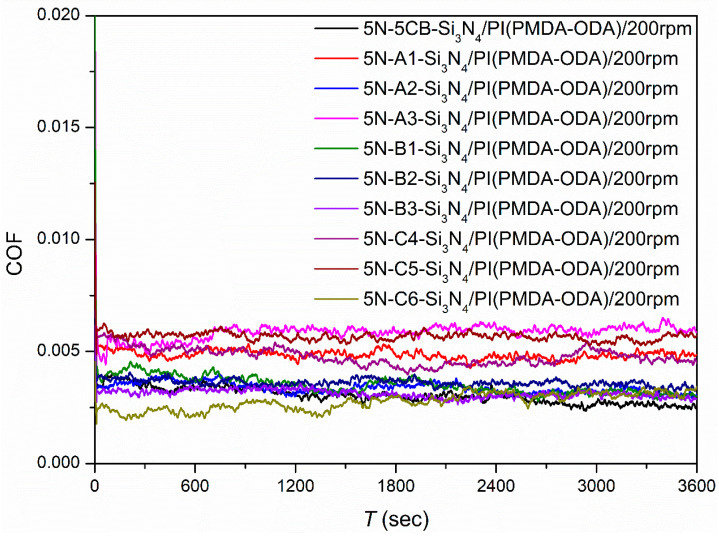
Friction coefficients as a function of time using the different LCs as the lubricant and Si_3_N_4_/PI as the friction pair.

Under a load of 5 N and a rotation speed of 200 rpm (177.92 mm/s), the friction tests were conducted in the point–plane contact mode with Si_3_N_4_/PI (PMDA-ODA) as the friction pair. The series of lubricants contained a total of 10 types of LC samples, i.e., 5CB and LCs with 5CB as the main component. The friction coefficients for each test are shown in Figure 2.

The test results show that when lubricated with 10 kinds of LCs, the friction coefficients of the systems with Si_3_N_4_/PI as the friction pair were in the range of 0.003~0.006, the fluctuation of the friction coefficient values was small, and the friction systems were in the superlubricity state. Among them, the Si_3_N_4_/PI friction system showed the best friction-reducing performance with a friction coefficient of 0.00277 when the mixed LCs C6 underwent (90% 5CB–10% 2CEPPN) lubrication. Therefore, we used LCs C6 as the lubricant in subsequent systematic studies.

#### 3.1.2. Friction Tests with the Optimal Lubricant

The systematic friction tests were conducted on a system with LCs C6 as the lubricant and Si_3_N_4_/PI as the friction pair.

##### Point–Plane Contact

(a) Different loads

**Figure 3:**
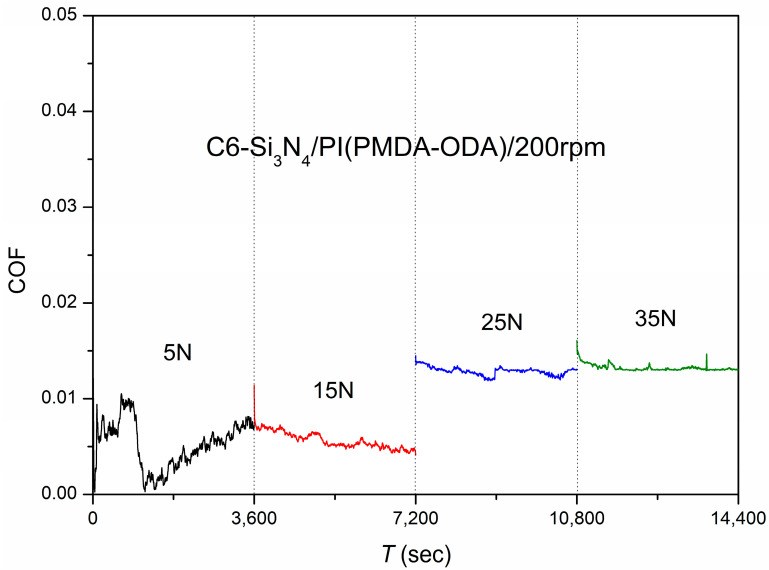
Friction coefficients as a function of time using C6 as the lubricant and Si_3_N_4_/PI as the friction pair with different loads.

The initial load for the test was 5 N at 200 rpm (177.92 mm/s). The test lasted 3600 s at each load, and then the load was increased by 10 N in the next cycle. The test was conducted until the PI film ruptured at a load of 45 N. Superlubricity occurred in this friction system at low loads (5 N and 15 N). When the load reached 25 N, the system was not in the process of superlubricity (the friction coefficient was 0.01288), indicating that the friction system was subjected to limited loads. The test results are shown in Figure 3.

(b) Different speeds

**Figure 4:**
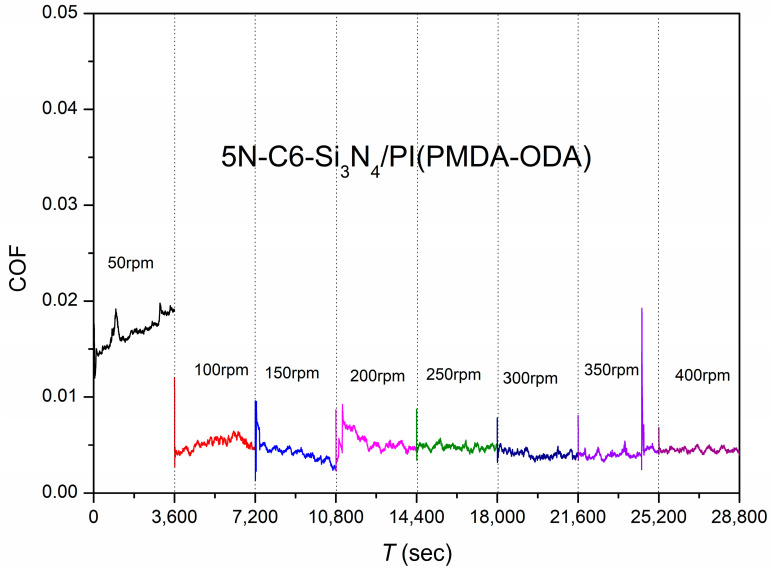
Friction coefficients as a function of time using C6 as the lubricant and Si_3_N_4_/PI as the friction pair at different speeds.

The test was carried out at a load of 5 N with an initial speed of 50 rpm (44.48 mm/s), each cycle ran for 3600 s, and the speed was increased by 50 rpm in the next cycle up to 400 rpm (355.84 mm/s). It was found that the system was in a stable superlubricity process at all speeds except at a low speed of 50 rpm, where the friction coefficient was 0.01689. The curve of friction coefficient versus the rotation speed for the system with C6 as the lubricant is shown in Figure 4.

(c) Long-time test

**Figure 5:**
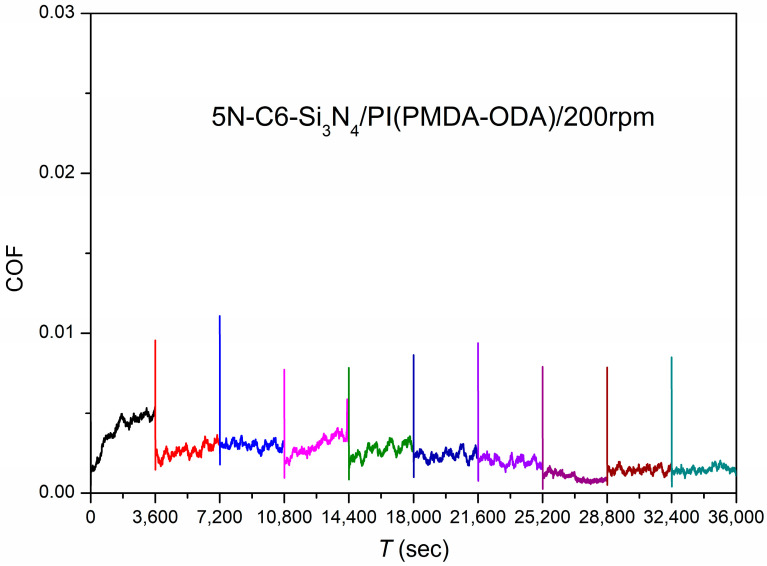
Friction coefficients as a function of time using C6 as the lubricant and Si_3_N_4_/PI as the friction pair in point–plane contact.

The test load was 5 N at 200 rpm with a test time of 3600 s per cycle. Ten cycles were performed to simulate the start–stop condition of the machine during operation. It was found that the friction system was in a stable state of superlubricity during the 10 test cycles, with the friction coefficient shown in Figure 5.

The friction test results of the system with LCs as the lubricant and Si_3_N_4_/PI as the friction pair in the point–plane contact mode illustrate that this type of superlubricity system has good repeatability and stability.

##### Plane–Plane Contact

**Figure 6:**
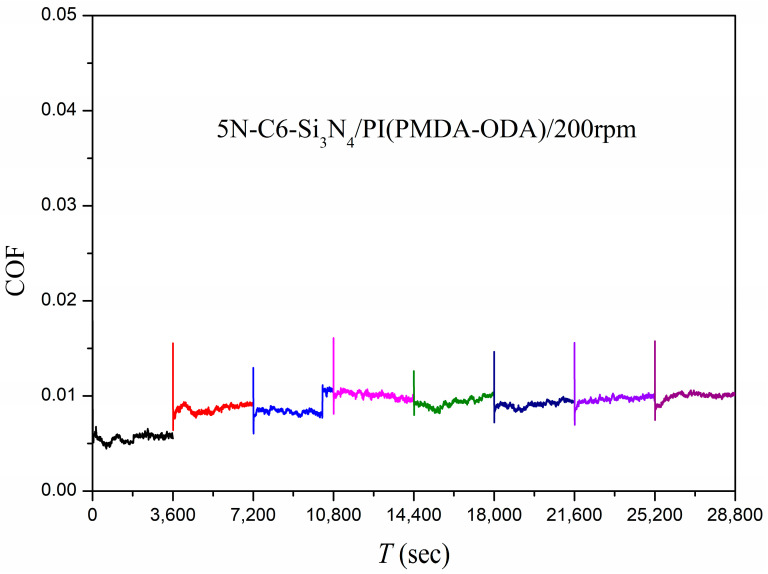
Friction coefficients as a function of time using C6 as the lubricant and Si_3_N_4_/PI as the friction pair in plane–plane contact.

The Si_3_N_4_ plane obtained by grinding was a circular plane with a diameter of about 2.15 mm. The Si_3_N_4_ plane was placed in face-to-face contact with the PI film at a test load of 5 N and a rotation speed of 200 rpm; the duration of each cycle was 3600 s, which still simulated the starting and stopping of the machine, and the machine could be stopped at the end of each cycle and then reloaded, running for eight consecutive cycles. The results of the friction coefficient for the system are shown in Figure 6. It was found that the system was in a superlubricity condition for almost the whole test, except for the fourth cycle, which slightly failed to reach the superlubricity condition (the friction coefficient of 0.01008). The result indicates that the stable superlubricity behavior of this system could be achieved in both point–plane or plane–plane contact modes.

### 3.2. GCr15/PI or SiC/PI Friction System

The mating part of the PI in the friction system was replaced with GCr15 steel or SiC, respectively, and plane–plane friction tests were conducted.

#### 3.2.1. GCr15/PI Friction System

**Figure 7:**
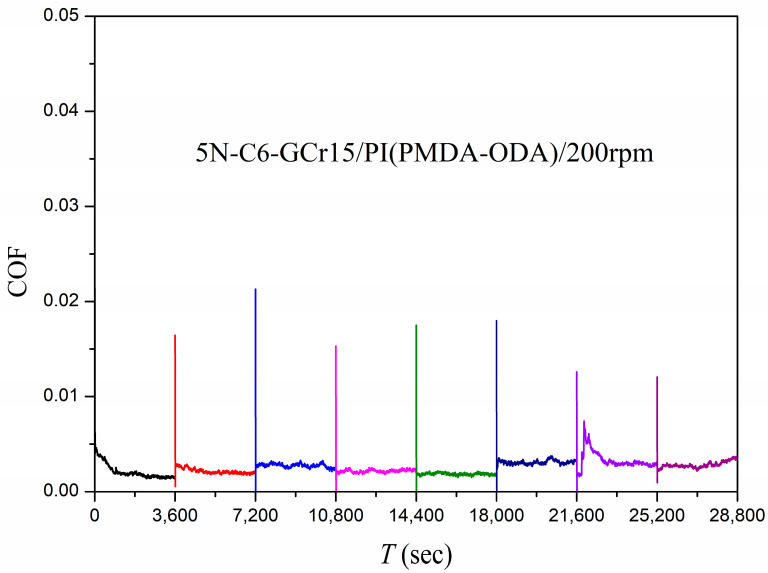
Friction coefficients as a function of time using C6 as the lubricant and GCr15/PI as the friction pair in plane–plane contact.

The plane of the GCr15 steel ball obtained by grinding was a circular plane with a diameter of about 1.79 mm. The plane–plane contact tests were performed on the GCr15 steel ball plane and the PI film to simulate the starting and stopping of the machine, and the results of the friction coefficient for the system after eight consecutive cycles are shown in Figure 7. The results show that this system was in a superlubricity condition throughout the test, which could be considered a very desirable superlubricity condition, with the average friction coefficient in the range of 0.00188–0.003312.

#### 3.2.2. SiC/PI Friction System

**Figure 8:**
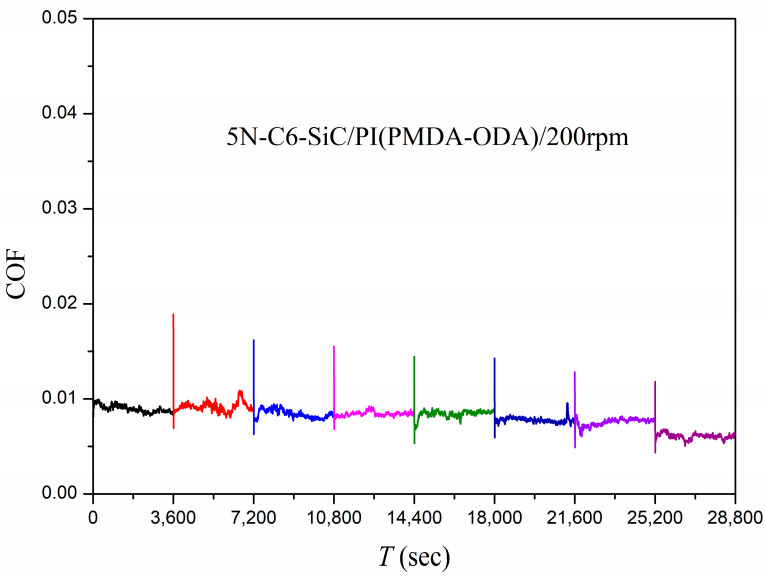
Friction coefficients as a function of time using C6 as the lubricant and SiC/PI as the friction pair in plane–plane contact.

The plane of the SiC ball, obtained by grinding, was a circular plane with a diameter of about 2.17 mm. The SiC ball plane and the PI film were subjected to face–face contact and a friction test, and the test results are shown in Figure 8. It was found that when the counterpart of the PI was SiC, the system was also in a stable superlubricity state during the eight test cycles.

In a friction system, the counterpart of PI can be Si_3_N_4_, GCr15 or SiC, respectively. As long as there are two friction elements, one of which is PI and the other LC, such a friction system can still exhibit superlubricity phenomena. The friction elements PI and LCs are the key factors in the occurrence of superlubricity in such friction systems. The counterpart of PI is the carrier of the friction behavior, and the specific material structure might have a small influence on superlubricity behavior.

### 3.3. Surface Analysis of PI

#### 3.3.1. Surface Topography Analysis

The surface morphology of the PI film after the friction test was observed using a white light interference microscope (Table 3), and it was found that when the counterpart was GCr15 steel, both 2D and 3D images showed some tiny surface structures. These results suggest that the friction coefficient of the system was minimized when the counterpart was GCr15 steel under the same test conditions. We wonder if the minimal friction coefficient is somehow related to the particular surface microstructure of the PI surface. We aim to conduct follow-up studies, such as generating microsurface structures on the PI surface, to investigate the correlation between friction properties and microsurface structures. The surface weaving-like phenomenon could reinforce a reduction in the friction coefficient, and the friction system showed a more significant superlubricity phenomenon, which is a very interesting experimental phenomenon. Moreover, we investigated the reasons for the appearance of such microstructures on the PI surface after rubbing when the counterpart was GCr15 steel and the reasons for the small amount or even absence of such microstructures on the PI surface after rubbing when the counterpart was ceramic.

#### 3.3.2. Surface Molecular Structure Analysis

We performed the silver mirror tests on the PI films that had been subjected to the plane–plane friction tests and on the PI films that had not been subjected to such tests. Since the number of LCs used in the friction test was very small and almost entirely diluted into the solution for the silver mirror test, the LCs used as a lubricant did not affect the results of the SERS test.

**Figure 9:**
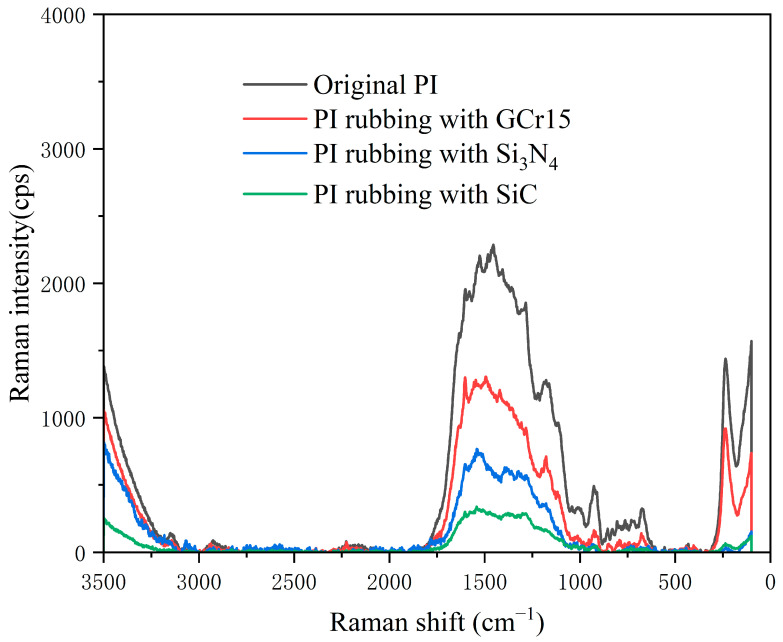
SERS spectra of PI film before rubbing and after rubbing.

Figure 9 shows the SERS spectra of PI films before and after friction. The positions of the peaks in the spectra of PI before and after friction were almost unchanged, indicating that friction was not strong enough to destroy the molecular structure of PI. However, SERS analysis clearly showed that the intensity of some peaks of the un-rubbing (original) PI was significantly higher than that of the rubbing PI. The SERS selection rule for molecules absorbed on metal surfaces is that molecular vibrations involving motions perpendicular to the surface should be enhanced in the spectra, while those involving motions parallel to the surface were weakened [39,40]. This indicates that some functional groups of PI change from perpendicular to parallel on the surface after friction. A comparison of the PI spectra before and after friction revealed that the intensities of the C=C backbone stretching vibrations of the benzene ring at 1600 cm^−1^ and 1450 cm^−1^, as well as the C-O-C stretching vibrations at 1280 cm^−1^ and 1177 cm^−1^, significantly changed. These results illustrate that the friction process greatly affected the surface orientation distribution of PI molecules, and some groups on the main and branched chains of PI molecules were parallel to the PI surface after friction. The PI material chosen for this work was from DuPont Kapton, which has a pretilt angle of about 1.0° with a mixture of fluorinated LCs [41], indicating that the oriented PI induced the LC molecules to undergo orientation almost parallel to the PI plane as well.

The SERS spectra reveal that friction can orient the PI molecules, and there is no specific requirement for the material that generates the friction, although the degree of orientation achieved by the PI molecules varies depending on the friction between the different materials and PI. The reasons for the varying degrees of orientation will also be the focus of our subsequent research. Friction causes the PI molecules to align, and the aligned PI induces the LC molecules to orient. The directional arrangement of LC molecules reminds us of an interesting and simple negative feedback mechanism in nature, the “Fish swarm effect”: a specific self-organization of fish swarms [36]. We hypothesize that this friction process is actually a free movement causing the orientation between LC molecules like a fish swarm guided by a negative feedback mechanism (stronger interaction forces between LC and PI and weaker interaction forces between LCs). Moreover, the pretilt angle of PI is only about 1.0°; the LCs are also arranged almost parallel to the PI surface, with the one-dimensional-ordered arrangement of LCs and very low viscosity so that the friction system occurs with superlubricity. The superlubricity mechanism of the system with Si_3_N_4_/PI as the friction pair and nematic LCs as the lubricant is similar to that of GCr15/PI as the friction pair, both of which maintained superlubricity by the PI-induced alignment mechanism.

## 4. Conclusions

For the occurrence of the superlubricity phenomenon involving PI, the alignment of PI by rubbing is the key factor leading to superlubricity. LCs are also important as lubricants because LC molecules can be induced to orient by aligning with PI as well. The selection of PI materials with small pretilt angles is also necessary for the occurrence of superlubricity. Interestingly, there are multiple PI counterparts available in this type of superlubricity system. The microstructure of the PI surface has an effect on the friction behavior of the system, and the next step should be to artificially design the surface microstructure for further study. PI is an important material for the production of bearings. Understanding the process and mechanism of the superlubricity phenomenon when PI is used as a friction component could provide experimental data for the application of PI as a production and processing equipment.

## Figures and Tables

**Table 1:** LC molecular structure formula.

Abbreviation of LC Name	Molecular Formula
5CB	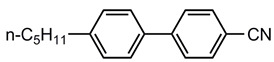
3UTPP2	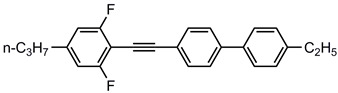
3UTPP4	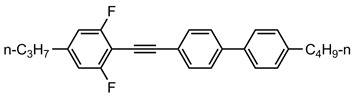
4UTPP3	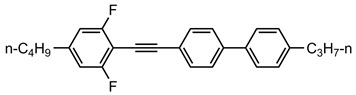
5CEPO2	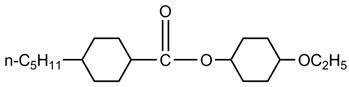
3CEPC3	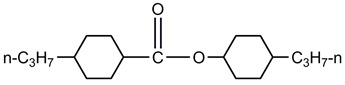
2CEPPN	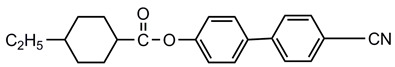

**Table 2:** Composition of mixed LCs.

LC Name	Base Composition	Base Ratio (wt.%)	Minor Component	Minor Ratio (wt.%)
A1	5CB	80	3UTPP2	20
A2	5CB	80	3UTPP4	20
A3	5CB	80	4UTPP3	20
B1	5CB	85	3UTPP2	15
B2	5CB	85	3UTPP4	15
B3	5CB	85	4UTPP3	15
C4	5CB	90	5CEPO2	10
C5	5CB	90	3CEPC3	10
C6	5CB	90	2CEPPN	10

**Table 3:** White light interference microscope images of rubbing PI.

Friction Pair	2D Image	3D Image
Si_3_N_4_/PI	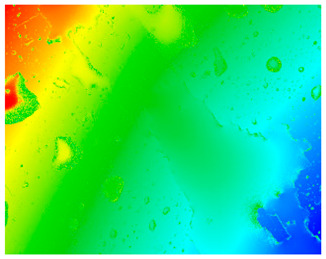	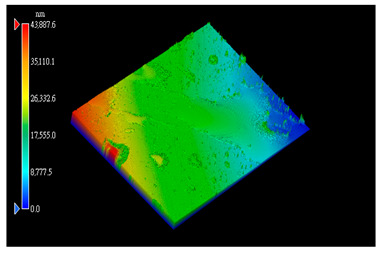
GCr15/PI	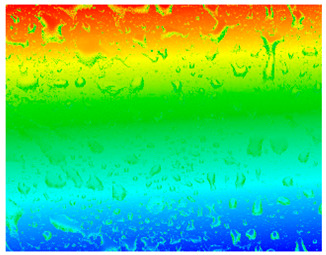	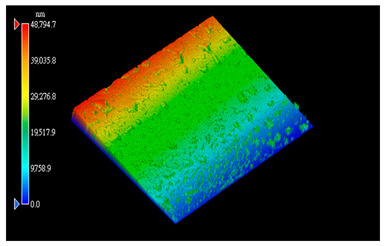
SiC/PI	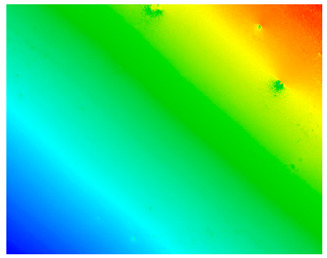	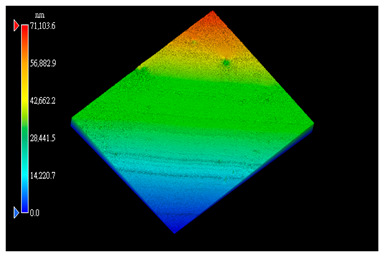

## Data Availability

Not applicable.
